# Dynamic profiling of amino acid transport and metabolism in Chinese hamster ovary cell culture

**DOI:** 10.1186/1753-6561-7-S6-P97

**Published:** 2013-12-04

**Authors:** Sarantos Kyriakopoulos, Karen M Polizzi, Cleo Kontoravdi

**Affiliations:** 1 Centre for Process Systems Engineering, Department of Chemical Engineering and Chemical Technology, Imperial College London, UK; 2 Division of Molecular Biosciences, Imperial College London, UK; 3 Centre for Synthetic Biology and Innovation, Imperial College London, UK

## Introduction

Chinese Hamster Ovary (CHO) cells are the most widely used industrial hosts for the production of recombinant DNA technology drugs [1]. In such processes amino acids (a.a.) are vital nutrients for growth, but also building blocks of the recombinant protein (rprotein). Our research aims to establish a better understanding of a.a. transport in and out of cells, since this could have significant impact on increasing productivity and designing feeding strategies during bioprocessing.

There are about 46 a.a. transporter proteins in mammalian cells, the genes of which are presented in Table 1 along with their substrates and all are members of the *Solute Carriers *(SLC) database [2]. A.a. transporters are subject to different expression profiles among mammalian cells and are grouped into more than 18 systems, based on sequence homology and function.

**Table 1:** Amino acid transporter genes based on the SLC database [2].

System	GENES	Substrates	Expresion/ Type of regulation	System	GENES	Substrates	Expresion/ Type of regulation
**A**	SLC38a1	Ala, Asn, Cys, Gln, His, Ser	below detection limits	**PAT**	SLC36a1	Gly, Ala, Pro, β- Ala, Tau	remains stable
	SLC38a2	Ala, Asn, Cys, Gln, Gly, His, Met, Pro, Ser	between cell lines^b^		SLC36a2	Gly, Ala, Pro	low^a^
	SLC38a4	Ala, Asn, Cys, Gly, Ser, Thr	within cell culture^c^		SLC36a3	putative	low^a^
				
**ASC**	SLC1a4	Ala, Ser, Cys, Thr	within cell culture^c^		SLC36a4	Ala, Pro, Trp	remains stable
				
	SLC1a5	Ala, Ser, Cys, Thr, Gln, Asn	both^d^	**T**	SLC16a10	Phe, Tyr, Trp	low^a^

**asc**	SLC7a10/ SLC3a2	Ala, Cys, Gly, Ser, Thr	low^a^	**X^-^_AG _**	SLC1a1	Asp, Glu	low^a^
				
**B^0 ^**	SLC6a19	Pro, Leu, Val, Ile, Met	low^a^		SLC1a2	Asp, Glu	both^d^
				
	SLC6a15	Pro, Leu, Val, Ile, Met	remains stable		SLC1a3	Asp, Glu	between cell lines^b^
				
**B^0,+ ^**	SLC6a14	basic & neutral a.a.	not checked		SLC1a6	Asp, Glu	below detection limits
				
**b^0,+ ^**	SLC7a9/ SLC3a1	Arg, Lys, Cystine	low^a^		SLC1a7	Asp, Glu	below detection limits

**β**	SLC6a6	Tau, β-Ala	both^d^	**x^-^_c _**	SLC7a11/ SLC3a2	Glu, Cystine	within cell culture^c^

**Gly**	SLC6a9	Gly	within cell culture^c^	**y^+ ^**	SLC7a1	Arg, Lys, His	both^d^
	SLC6a5	Gly	low^a^		SLC7a2	Arg, Lys, His	low^a^
	SLC6a18	Gly	below detection limits		SLC7a3	Arg, Lys, His	low^a^

**IMINO**	SLC6a20	Pro	low^a^	**y^+^L**	SLC7a7/ SLC3a2	Lys, Arg, Gln, His, Leu, Met	both^d^
				
**L**	SLC7a5/ SLC3a2	Cys, Leu, Phe, Trp, Val, Tyr, Ile, His, Met	both^d^		SLC7a6/ SLC3a2	Lys, Arg, Gln, His, Leu, Met, Ala, Cys	remains stable
				
	SLC7a8/ SLC3a2	neutral a.a., except Pro	low^a^	**His & small peptides**	SLC15a3	His	between cell lines^b^
				
	SLC43a1	Leu, Ile, Met, Phe	low^a^		SLC15a4	His	between cell lines^b^
	SLC43a2	Leu, Ile, Met, Phe	between cell lines^b^	**Heavy subunits of hetero-meric**	SLC3a1	various based on "partner"	low^a^
	SLC43a3	putative	between cell lines^b^		SLC3a2	various based on "partner"	both^d^

**N**	SLC38a3	Ala, Asn, Gln, His	not checked	**Not in a system**	SLC6a7	Pro	not checked
	SLC38a5	Gln, Asn, His, Ser	both^d^		SLC6a17	neutral a.a.	not checked
				
					SLC7a13	Asp, Glu	not checked
					SLC12A8	putative	not checked

The "Expression/ Type of regulation" column refers to our results for the CHO cell lines described in the materials & methods section: ^a^low levels-refers to fractional copies per cell; ^b^regulation between cell lines-refers to regulation significantly higher than two fold at least at a time point between the different cell lines presented; ^c^regulation within cell culture-refers to differential expression (significantly higher than two fold) at least at a time point within cell culture of a given cell line; ^d^both types of regulation-refers to a gene presenting both ^b ^and ^c ^as discussed previously.

To our knowledge, there is no comprehensive study of a.a. transporters in industrially relevant CHO cells in the literature. To that direction, a.a. transporter genes were profiled during batch culture of three CHO cell lines with varying levels of productivity. In parallel, the intra- and extracellular levels of a.a. were quantified.

## Materials and methods

Three cell lines were kindly donated by Lonza Biologics. GSn8 cell line was transfected with an empty glutamine synthetase (GS) vector. GS35 and GS46 cell lines were both transfected with a GS vector that also carries the heavy and light chains of a chimeric IgG4 antibody. The specific productivity of cell line GS46, quantified by a commercial ELISA kit (Bethyl laboratories, US), is approximately double that of GS35 one.

Batch cultures were performed in triplicate in 1L Erlenmeyer flasks with a working volume of 300mL in CD-CHO medium (Invitrogen, UK) supplemented with 25 μM MSX (Sigma, UK). Viable cell concentration was determined daily using the trypan blue dye exclusion method.

40 a.a. transporters were studied in all cell lines using real time quantitative reverse transcription polymerase chain reaction on samples from different phases of batch culture. Samples were collected at day 4 (exponential phase) and day 6 & day 7 (stationary phase) of the growth curve for all cell lines (samples were also taken at day 3 for IgG4 producers only and day 9 for the null cell line only). Results are reported against the housekeeping gene "actb". Housekeeping genes "vezt" and "hirip3" were also well correlated.

The extracellular and intracellular a.a. profiles were monitored daily using high performance liquid chromatography (PicoTag, Waters, UK). Intracellular samples were quenched with 0.9% w/v NaCl and extracted with a 50% aqueous acetonitrile solution, as described in [3].

## Results

The results (Table 1) reveal that ~30% of transporters are lowly expressed (fractional copies per cell), 9% are below levels of detection, whereas 40% are significantly differentially expressed either during batch cell culture, or between cell lines, or both. The remaining transporters appear to remain stable.

### Regulation within culture

**Figure 1:**
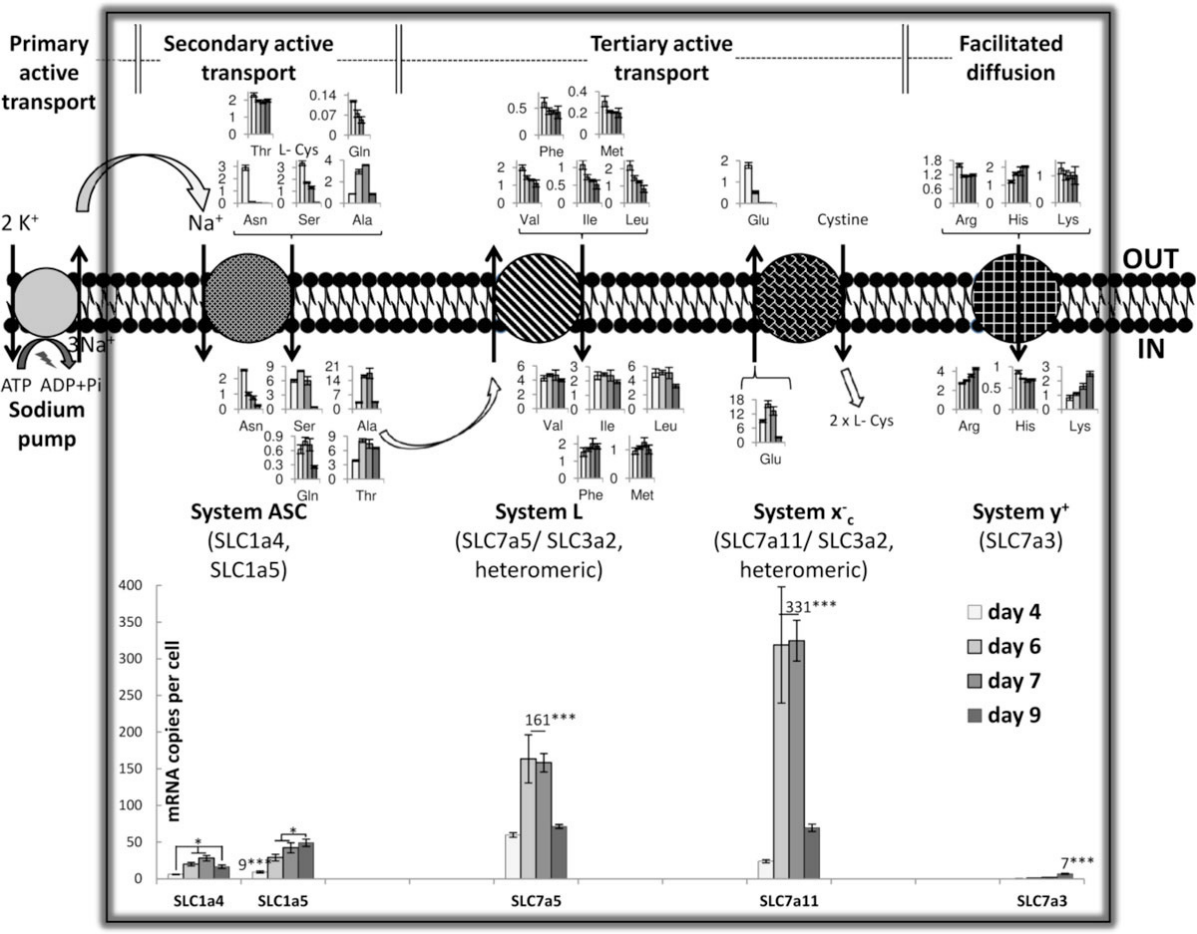
**A map associating the differentially expressed amino acid transporters for the null cell line, their amino acid substrates, and the intracellular concentrations (femtomol/ cell, in the area designated by the "IN" tag) and extracellular concentrations (mM, in the area designated by the "OUT" tag) of the latter**. A.a. transport is highlighted by the black box. The expression of the mRNA levels of the differentially expressed a.a. transporters (in mRNA copies per cell) at different phases of cell culture, exponential (day 4), stationary (days 6 & 7), and decline (day 9) is displayed at the bottom, where stationary phase samples are averaged, since not statistically different (for ease of statistical analysis visualization). The relevant energy utilisation mechanisms of each system are also depicted (top). Genes: slc6a9 (glycine), slc1a2 (acidic a.a.), slc7a7 (basic and branched chain a.a.) and its heteromeric transporter slc3a2 were also found to be differentially expressed, but are not presented in this figure. Our chosen a.a. analysis method was not able to quantify cysteine (L-Cys) levels.

The majority of the transporters are found to be upregulated at stationary phase for all cell lines, as also presented in Figure 1, where a mapping of a.a. metabolism and transport has been illustrated for the null cell line. Specifically, five genes encoding for transporters of a.a. relating to the glutathione (GSH) pathway were found to be upregulated significantly higher than 2 fold at stationary phase, when compared to exponential phase for all cell lines. These genes were: slc1a4 (Ala and Cys), slc6a9 (Gly), slc1a2 (Glu and Asp), slc7a11 (Cystine and Glu), and heteromeric transporter slc3a2 which partners with slc7a11. GSH is a well-known marker of oxidative stress [4], high levels of which have been associated with high productivity [5].

### Regulation between cell lines

In their majority, genes were found to be upregulated for protein producing cell lines at all time points. Genes whose expression is upregulated significantly (two-fold or higher) in the protein-producers at all time points analyzed were: slc43a2 (system L, leucine and branched-chain a.a.) and slc1a2 (system X^-^_AG, _glutamate and aspartate). However, no genes, apart from slc6a6 (taurine and b-Ala), were found to be differentially expressed between high (GS46) and low producer (GS35). We find slc6a6 gene differentially expressed early in cell culture (day 3), which makes us hypothesize that the gene could be a candidate for selection purposes. The overexpression of this gene in CHO cells has been found to significantly enhance growth and productivity [6].

### Feeding strategy based on order of feeding

The a.a. transporters gene expression findings correlate well with the extracellular and intracellular concentration profiles of their respective substrates (Figure 1). By analysing the differentially expressed genes for a specific cell line a feeding strategy can be designed. For example, we find transporter slc7a5, of system L, highly upregulated at stationary phase for the null cell line (Figure 1). This transporter exchanges an intracellular neutral a.a. with an extracellular branched chain one (isoleucine, leucine, valine). Branched chain amino acids are associated with the mTor signalling pathway, essential regulator for many physiological roles in mammalian cells [7]. Hence, a feeding strategy can be proposed, where neutral amino acids are fed first and followed by branched chain amino acids, in order for them to be more effectively uptaken. A similar type of pre-conditioning was found to significantly enhance cellular protein production in another type of mammalian cells [7].

## Conclusions

Glutathione pathway associated a.a. transporters (slc1a2, slc1a4, slc6a9, slc7a11/ slc3a2) can be targeted as genetic engineering targets, since are all found highly upregulated at stationary phase of cell culture. Additionally, transporters slc1a2, slc43a2 are associated with rprotein productivity, since all of them are found to be upregulated for producing cell lines vs the null. Gene slc6a6, carrying taurine and β-alanine, can be associated with high productivity (as also suggested in [6]), as was also found to be differentially expressed in the high vs the low producer early in cell culture. A feeding strategy can be proposed, based on our results that remains to be tested experimentally. Finally, extending this integrative approach to the proteome level would help link regulation at the transcriptomic level to actual differences in transport capability.
